# Supplementary material for: Persistent Hypogammaglobulinemia after Receiving Rituximab Post-HSCT Is Not Caused by an Intrinsic B Cell Defect
Source: Int J Mol Sci. 2023 Nov 6;24(21):16012. doi: 10.3390/ijms242116012 (PMC10649739; doi:10.3390/ijms242116012)
Supplement: Supplementary file 1 [file ijms-24-16012-s001.zip › ijms-2688735-supplementary.pdf]

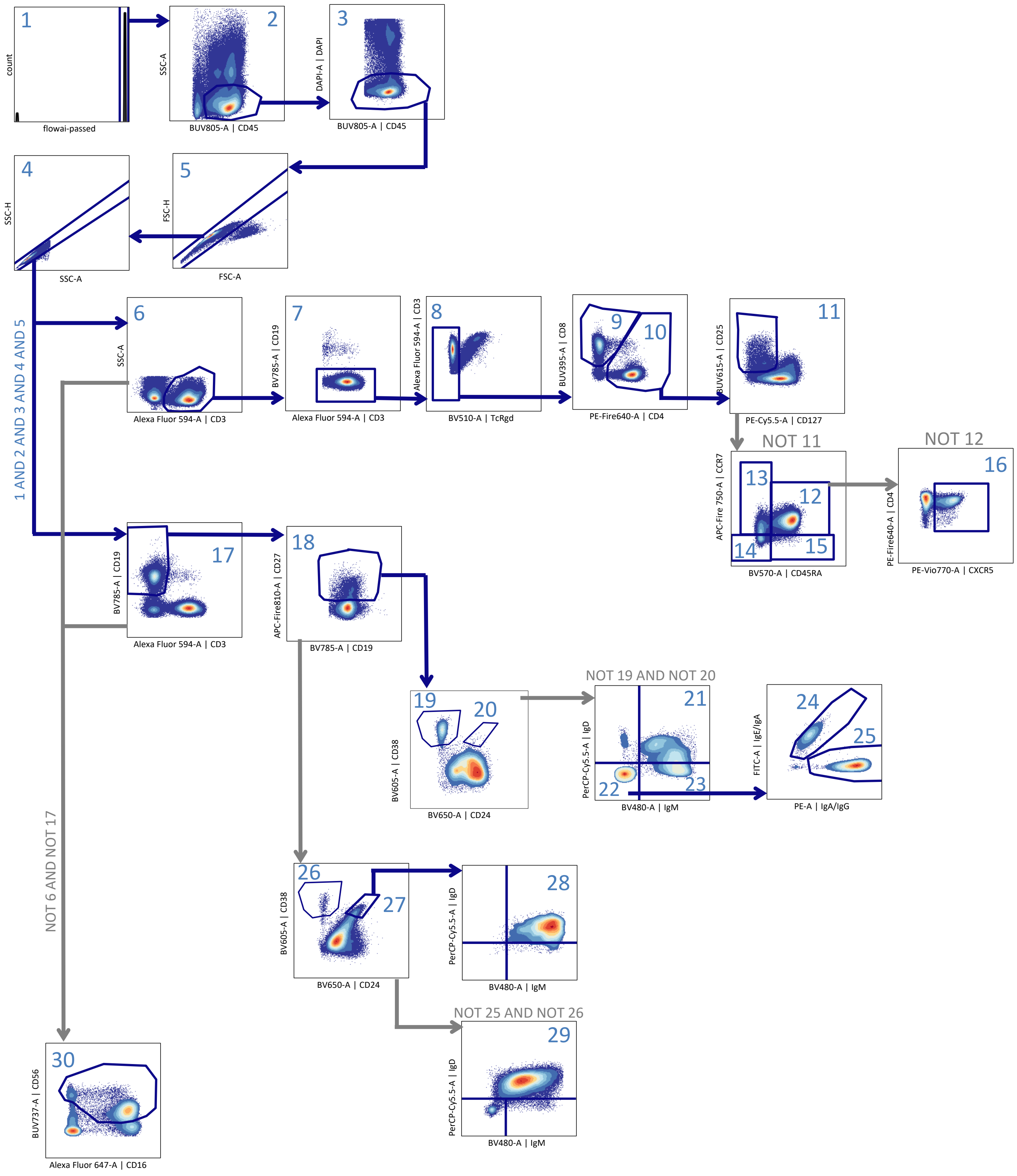

Figure S1. Gating strategy: (1-5) high quality live single events; (7) T-cells; (9) CD8+ TcRgd- T-cells; (10) CD4+ T-cells; (11) CD4+ T regulatory cells; (12) CD4+ naïve T-cells; (13) CD4+ central memory T-cells; (14) CD4+ effector memory T-cells; (15) CD4+CD45RA+ effector memory T-cells; (16) Tfh like memory T-cells; (17) B-cells; (19) plasmablasts; (21) Marginal zone like/effector/ unswitched memory B-cells; (22+23) memory B-cells; (23) memory B-cells IgM+ only; (24) IgA+ switched memory B-cells; (25) IgG+ switched memory B-cells; (28) transitional B-cells; (29) naïve mature B-cells; (30) NK-cells.

Table S1. Flow Cytometry antibodies used.

CD CODE	TARGET	CLONE ID	LABEL	SUPPLIER
CD3		UCHT1	AF594	Biolegend
CD4		SK3	PE-Fire640	Biolegend
CD8		RPA-T8	BUV395	BD Biosciences
CD16		3G8	AF647	Biolegend
CD19		SJ25C1	BV786	BD Biosciences
CD24		ML5	BV650	BD Biosciences
CD25		2A3	BUV615	BD Biosciences
CD27		QA17A18	APC-Fire810	Biolegend
CD38		HIT2	BV605	Biolegend
CD45		HI30	BUV805	BD Biosciences
CD45RA		HI100	BV570	Biolegend
CD56		NCAM16.2	BUV737	BD Biosciences
CD127	IL-7Ralpha	eBioRDR5	PE-Cy5.5	eBioscience
CD185	CXCR5	REA103	PE-Vio770	Miltenyi biotec
CD197	CCR7	G043H7	APC-Fire750	Biolegend
	IgA	IS11-8E10	FITC	Miltenyi biotec
	IgA	IS11-8E10	PE	Miltenyi biotec
	IgD	IA6-2	PerCP-Cy5.5	Biolegend
	IgG	G18-145	PE	BD Biosciences
	IgM	G20-127	BV480	BD Biosciences
	TCRgd	11F2	BV510	BD Biosciences
